# Synergistic Incorporation of Boron Nitride Nanosheets and Fluoropolymers to Amplify Anti-Corrosion Attributes of Waterborne Epoxy Resin

**DOI:** 10.3390/polym17081020

**Published:** 2025-04-10

**Authors:** Hui Ma, Xuan Liu, Xiaofeng Han, Rui Yang, Zhaotie Liu, Jian Lv

**Affiliations:** 1 Department of Chemistry and Chemical Engineering, Shaanxi University of Science and Technology, Xi’an 710021, China; huima_204@163.com (H.M.); liuxuan@sust.edu.cn (X.L.); 210812155@sust.edu.cn (X.H.); y102247@163.com (R.Y.); 2 State Key Laboratory of Fluorine &; Nitrogen Chemicals, Xi’an Modern Chemistry Research Institute, Xi’an 710065, China

**Keywords:** waterborne epoxy resin, boron nitride nanosheets, fluoropolymers, electrochemical impedance spectroscopy

## Abstract

The corrosion of metal substrates is closely associated with the permeability of the corrosive medium in which they are immersed. To enhance the protection of metal materials and improve anti-corrosion performance from an epoxy resin perspective, the diffusion path complexity can be increased and porosity reduced within the epoxy resin coating to effectively block the invasion of corrosive media. Simultaneously, reducing the affinity between the corrosive media and the epoxy resin coating makes it difficult for corrosive substances to adhere. Based on this principle, this study introduces two-dimensional boron nitride nanosheets (BNNS) and fluoropolymers-modified one-dimensional nano-silica (SiO_2_) and organic tannic acid as fillers to jointly enhance the protective effect of waterborne epoxy-resin-based composites. Experimental results demonstrate that when the BNNS content is 0.5 wt.%, the 0.5-BNNS/WEP composite coating exhibits superior anti-corrosion performance, achieving an electrochemical impedance of 2.90 × 10^7^ Ω∙cm^2^. Moreover, when BNNS is compounded with fluorinated SiO_2_ or fluorinated tannic acid as fillers and incorporated into waterborne epoxy resin, the resulting composite coatings maintain excellent long-term anti-corrosion performance even after 20 days of salt spray testing.

## 1. Introduction

Corrosion not only impedes the normal utilization of metallic materials but also accelerates their degradation, leading to substantial economic losses and potential casualties [1]. Consequently, the development of effective anti-corrosion technologies is of paramount importance. Presently, the predominant anti-corrosion methods encompass organic coatings [2,3], corrosion inhibitors [4,5], and cathodic protection [6]. Among these, organic coatings are particularly favored due to their low cost, ease of application, and favorable mechanical properties. Specifically, waterborne epoxy resin (WEP) coatings have gained widespread use in anti-corrosion primers and industrial flooring applications owing to their environmental compatibility and superior adhesion [7]. However, the protective efficacy of epoxy resin coatings remains suboptimal. Microcracks and pores that form during the curing process facilitate the ingress of corrosive media such as water, oxygen, and ions, thereby diminishing the anti-corrosion performance [8]. To address these issues, current modification strategies include the incorporation of nanoparticles [9,10], superhydrophobic surface treatments [11,12], the addition of corrosion inhibitors nanocapsules [13], the design of novel molecular structures [14], and the development of self-healing multifunctional composite coatings [2].

Two-dimensional layered materials, including graphene oxide (GO) [6,15], molybdenum disulfide (MoS_2_) [16], and hexagonal boron nitride (h-BN) [17,18], have emerged as prominent candidates for anti-corrosion coatings due to their large specific surface areas, which effectively inhibit the penetration of corrosive media such as water, chloride ions (Cl^−^), and oxygen. Boron nitride nanosheets (BNNS), often referred to as “white graphene”, are distinctive two-dimensional nanofillers characterized by environmental friendliness, high surface area, superior chemical and mechanical strength, thermal and electrical stability, and corrosion resistance [4,19,20,21]. As a graphene-like material, BNNS exhibits remarkable impermeability, chemical inertness, and thermal stability, thereby preventing galvanic corrosion owing to its insulating properties. Compared with zero-dimensional and one-dimensional nanomaterials, BNNS demonstrates enhanced impermeability and a larger specific surface area. When incorporated into epoxy resin (WEP) coatings, BNNS significantly enhances long-term durability by extending diffusion paths and leveraging the “labyrinth effect” to impede the ingress of water molecules, corrosive ions, and oxygen to the metal surface. However, the significant electronegativity difference between B and N atoms in h-BN results in strong interlayer interactions within BNNS layers, leading to aggregation. This aggregation disrupts the integrity of the coating and diminishes its protective efficiency. Cui et al. [22] addressed this issue by performing non-covalent functionalization of h-BN using oligomers, which significantly improved its dispersibility in organic solvents. Experimental results demonstrated that the low-frequency impedance modulus of the epoxy resin composite coating containing 1.0 wt.% modified h-BN was enhanced by three orders of magnitude relative to the pure epoxy coating. Liao et al. developed 5-aminotetrazole@ZIF-7@zinc gluconate/BNNS [20] and amino-coated carbon dots/BNNS [19] as functional nanofillers, enhancing the durability of the WEP coating by 1–1.5 orders of magnitude. Furthermore, they utilized 2-amino-5-mercapto-1,3,4-thiadiazole (ATT) as an intercalating agent to successfully exfoliate BNNS and prepare ATT/BNNS composite nanofillers [4]. The resulting coating, formed by combining these composite nanofillers with WEP, demonstrated exceptional long-term anti-corrosion performance. Specifically, after 40 days of immersion in a 3.5 wt.% NaCl solution, its impedance modulus at 0.01 Hz was 100 times higher than that of the pure WEP coating. However, while modified BNNS alone provides effective physical shielding, it does not adequately prevent electrochemical corrosion.

In addition to enhancing the tortuosity of the diffusion path of the corrosive medium, reducing its affinity with the epoxy resin coating is also an effective strategy to improve the anti-corrosion performance of waterborne epoxy resins. Due to their low surface energy, fluoropolymers are frequently employed for hydrophobic modification [11,23,24]. Guan et al. [25] successfully developed a superhydrophobic composite coating composed of 1H, 1H, 2H, 2H-perfluorodecyltriethoxysilane (PFDS), ethylenediaminetetraacetic acid (EDTA), and anodic copper oxide (ACO) on the surface of copper alloys. This coating exhibited remarkable superhydrophobic properties, achieving a water contact angle of up to 164.53°. Moreover, the micro-nano structure of the coating can trap a gas film, significantly reducing the contact area between the coating and the corrosive solution, thereby effectively inhibiting the interaction among scaling crystals, the corrosive medium, and the substrate, thus further enhancing the anti-scaling and anti-corrosion capabilities of the coating. Wang et al. [24] synthesized a novel high-performance hydrophobic fluorine-substituted polyaniline (PFAN) filler. Experimental results indicated that this filler not only passivates iron but also effectively prevents electrolyte penetration. These characteristics enabled the epoxy coating containing 3 wt.% PFAN to exhibit excellent long-term anti-corrosion performance. The composite materials of polymer and nanomaterials enhance the mechanical properties of the polymer matrix, improve the pore size distribution of the matrix, and affect the electrical conductivity [26].

Considering the unique properties of boron nitride nanosheets (BNNS) and fluoropolymers, this study first utilized a synergistic effect of two surfactants to exfoliate hexagonal boron nitride (h-BN). The BNNS were incorporated as fillers into WEP, and the effect of varying filler concentrations on the anti-corrosion performance of the composite coating was evaluated. Additionally, SiO_2_ and tannic acid were polymerized with hexafluorobutyl acrylate via free radical polymerization to produce fluorinated SiO_2_ (FSiO_2_) and fluorinated tannic acid (FTA), respectively. Furthermore, BNNS were combined with FSiO_2_ or FTA (BFS or BFT) and dispersed in WEP to significantly enhance their anti-corrosion properties.

## 2. Materials and Methods

### 2.1. Materials

Tannic acid (TA, Aladdin, AR, Shanghai, China), hexagonal boron nitride (h-BN, Aladdin, 99.9%), toluene (Macklin, AR, Shanghai, China), hydrophilic nano-silica (SiO_2_), 3-(isobutenoyloxy) propyl trimethoxysilane (KH570, Macklin, 98%), triphenylphosphine (TPP, Macklin, >99%), glycidyl methacrylate (GMA, Macklin, 98%), hexafluorobutyl acrylate (HFBA, Macklin, 98%), tetrahydrofuran (THF, Macklin, 99%), *N,N’*-dimethylformamide (DMF, Macklin, AR), cetyltrimethylammonium bromide (CTAB, Macklin, 99%), sodium dodecyl sulfate (SDS, Macklin, 99%), ethanol (Macklin, AR), and Q235 steel plates (Macklin) were used as received without undergoing additional purification processes. First, 2,2-Azobisisobutyronitrile (AIBN, Aladdin, 98%) was subjected to three rounds of recrystallization using methanol and subsequently dried under vacuum conditions. Waterborne epoxy resin (WEP) was obtained from Wuxi Changgan Chemical Company Limited, Wuxi, China. The defoamer (902W), leveling agent (BYK333), and wetting agent (4100) were provided by Shaanxi Baotashan Paint Company Limited, Xingping, China. All materials were utilized as received, without undergoing additional purification.

### 2.2. Exfoliation of h-BN

First, 1 g of h-BN, 0.3 g of CTAB, and 0.3 g of SDS were added to a beaker containing 100 mL of deionized water and stirred for 1 h. The resulting h-BN suspension was subsequently transferred to an ultrasonic cell disruptor for sonication treatment lasting 8 h. Following this, the dispersion was centrifuged at 5000 rpm for 20 min. Finally, exfoliated boron nitride nanosheets (BNNS) were obtained by freeze-drying the supernatant.

### 2.3. Fabrication of Fluorinated Nanosilica (FSiO_2_) and Fluorinated Tannic Acid (FTA)

Preparation of fluorinated nanosilica (FSiO_2_) was conducted according to previously reported methods [27]. For the synthesis of FTA, 5.0 g (73 mmol) of TA and 0.52 g (2.0 mmol) of TPP were dissolved in 40 mL of THF and transferred to a 250 mL round-bottom flask. Thereafter, 21 g (147 mmol) of GMA was slowly added dropwise over a period of 30 min. The mixture was then refluxed and stirred at 90 °C under a nitrogen atmosphere for 20 h. Upon completion of the reaction, excess GMA and TPP were removed by precipitation with toluene, followed by the removal of unreacted TA through precipitation with water. The resulting product was vacuum-dried at room temperature for 48 h, ground into a fine powder, and stored in a cool, dry place. This material was designated as methacrylated TA (MTA). Finally, MTA was mixed with HFBA at a mass ratio of 0.5:100, with 1 wt.% AIBN added as an initiator. Using 20 mL of DMF as the solvent, the mixture was stirred and reacted at 75 °C under nitrogen protection for 10 h to prepare the FTA solution.

### 2.4. Preparation of BNNS/WEP, BFS/WEP and BFT/WEP Composite Coatings

The Q235 steel plate, measuring 60 mm × 130 mm with a thickness of 0.5 mm, was polished using 1000-mesh sandpaper. The surface was subsequently cleaned with absolute ethanol and allowed to air-dry at room temperature until completely dry for subsequent use. Different mass fractions of BNNS were sequentially mixed with 30 g of WEP at a rotational speed of 800 rpm for 30 min. Thereafter, the curing agent (F0750, 10 g), defoamer (902W, 0.5 g), leveling agent (BYK333, 0.5 g), and wetting agent 4100 (0.5 g) were added sequentially at 5-min intervals. Following thorough mixing, the solution was filtered through a 200-mesh filter cloth. The resultant coating was uniformly applied onto pretreated Q235 steel plates using a 150 μm magnetic rod. The coated plates were conditioned at ambient temperature for three days before being cured in an oven maintained at 60 °C for 24 h. After curing, the coated steel plates were cut into 60 mm × 1.5 mm strips and encapsulated with a wax mixture (rosin:paraffin = 1:1.5). For the preparation of BFS/WEP and BFT/WEP composite coatings, 0.1 wt.% FSiO_2_ or 4.9 mL FTA was mixed with 0.5 wt.% BNNS and 30 g WEP for 30 min under the same conditions, followed by identical processing steps.

## 3. Characterization

The morphology of h-BN before and after exfoliation and the cross-sectional morphology of the composite coatings were characterized by scanning electron microscope (SEM, FEI Verios, Waltham, MA, USA). The particle size distribution of BNNS was characterized using dynamic light scattering (DLS) with a Zetasizer Nano ZS instrument: BNNS was dispersed in absolute ethanol for 30 min under ultrasonic treatment. Subsequently, a small volume of the dispersion was transferred to a four-way cuvette. Measurements were conducted at a 90° scattering angle and 25 °C, with a 2-min equilibration period. Each experiment was repeated three times to ensure data reliability. Fourier-transform infrared spectroscopy (FT-IR, VECTOR-22, Nicolet, Madison, WI, USA) was employed to analyze BNNS, FSiO_2_, and FTA within the wavelength range of 4000 to 400 cm^−1^. An optical contact angle meter (DSA30) was utilized to measure the contact angle between the liquid and the surface of the composite coatings.

### 3.1. Evaluation of the Water Absorption Performance of the Composite Coating

Initially, the dried coating samples were accurately weighed to determine their initial mass, followed by complete immersion in deionized water. The samples were removed at specific intervals (0th, 5th, 10th, and 20th day) to record the mass changes. The coating’s water absorption rate was calculated based on the formula below:

(1) $$\mathrm{Water}\;\mathrm{absorption}=\frac{m_{2}-m_{1}}{m_{1}}\times 100\%$$

where *m*_1_ is the initial quality of the sample, *m*_2_ is the quality of the sample after water absorption.

### 3.2. Mechanical Property Testing of the Composite Coating

Dumbbell-shaped specimens with a length of 3 cm were prepared, and their thickness was accurately measured using a micrometer. Marking lines were made at the center of each specimen (i.e., at 1.5 cm) to ensure uniform force distribution during the tensile test, thereby enabling precise measurement of the stress–strain properties of the composite coating material.

### 3.3. Evaluate the Corrosion Resistance Characteristics of the Composite Coating

To assess the corrosion resistance of the composite coating, electrochemical impedance spectroscopy (EIS) testing was conducted in this study utilizing a three-electrode system. Specifically, a glassy carbon electrode with a diameter of 5 mm was utilized as the working electrode, a saturated calomel electrode functioned as the reference electrode, and a platinum wire with an area of 1 square centimeter served as the counter electrode. The electrolyte solution consisted of an aqueous medium containing 3.5% (*w/v*) sodium chloride. All tests were performed in an electromagnetic shielding environment, covering a frequency range of 10^−2^ to 10^5^ Hz with a voltage amplitude maintained at 20 mV. To ensure data accuracy, each sample underwent three parallel tests to minimize errors [28]. Additionally, salt spray resistance performance tests were conducted according to the Chinese national standard [29], and EIS tests on the composite coating were performed on days 0, 5, 10, and 20 [27].

## 4. Results and Discussions

### 4.1. Morphology of h-BN and BNNS and Particle Size Analysis of BNNS

**Figure 1:**
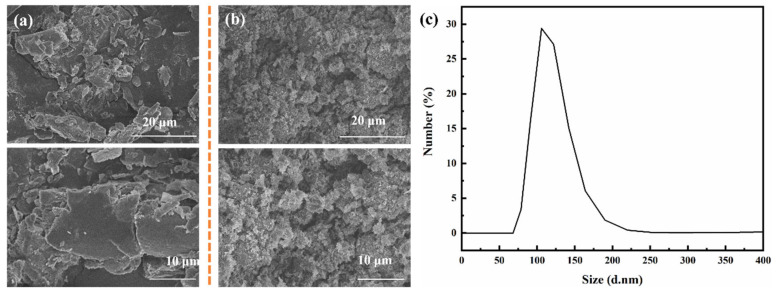
SEM morphology of h-BN (**a**) before and (**b**) after exfoliation, (**c**) particle size distribution of BNNS.

Scanning electron microscopy (SEM) was employed to characterize the h-BN samples before and after exfoliation. Figure 1 presents SEM images of h-BN (a) prior to exfoliation and BNNS (b) post-exfoliation. The SEM images reveal that the pre-exfoliated h-BN exhibits a lamellar structure with particle sizes ranging from 1 to 3 μm. Following an 8 h treatment in a cell crusher using CTAB and SDS, the BNNS were successfully prepared, resulting in a reduction of particle size to the nanometer level. Compared to pristine h-BN, exfoliated BNNS demonstrate pronounced surface, size, and quantum confinement effects due to their reduced dimensions. Furthermore, dynamic light scattering (DLS) analysis was conducted to measure the particle size distribution of the exfoliated BNNS sample, as illustrated in Figure 1c. The DLS results indicate that the particle size of the BNNS sample is primarily centered around 125 nm, thereby confirming the effectiveness of the cell crusher method combined with surfactants CTAB and SDS for achieving efficient h-BN exfoliation.

### 4.2. FT-IR Spectra and XRD Analysis of h-BN Before and After Exfoliation

**Figure 2:**
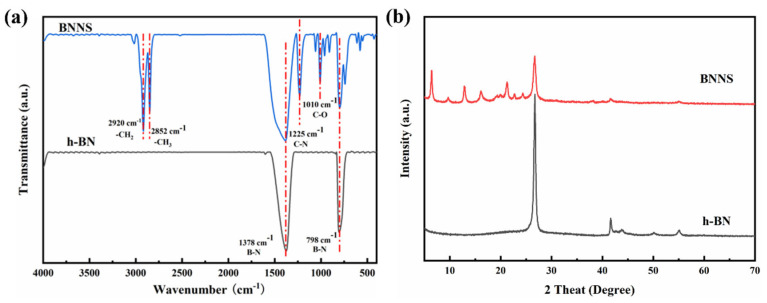
(**a**) FT-IR spectra and (**b**) XRD plots of h-BN before and after exfoliation.

These samples were characterized using Fourier-transform infrared spectroscopy (FTIR) within the wavenumber range of 4000 to 400 cm^−1^, and the results are presented in Figure 2a. Both pristine h-BN and exfoliated h-BN display prominent absorption peaks at 1378 cm^−1^ and 798 cm^−1^. These peaks are attributed to the in-plane stretching vibration and out-of-plane bending vibration of the B-N bonds, respectively [30,31]. Additionally, the FTIR spectrum of BNNS reveals characteristic peaks at 2920 cm^−1^ and 2852 cm^−1^, which are attributed to the stretching vibrations of CH_2_ and CH_3_ groups from CTAB and SDS. The peak at 1010 cm^−1^ is associated with the C-O bond stretching vibration in SDS, while the peak at 1125 cm^−1^ corresponds to the C-N bond stretching vibration in CTAB. These findings suggest that CTAB and SDS molecules adsorbed onto the surface of BNNS during the exfoliation process, thereby modifying its surface properties. The FT-IR spectra of FSiO_2_ and FTA (as shown in Figure S1) confirm the successful synthesis of the targeted fluorinated polymers through the presence of characteristic functional groups.

X-ray diffraction (XRD) measurements were performed to study the crystal structure characteristics of the prepared samples, and the results are presented in Figure 2b. The XRD pattern of pristine h-BN reveals its main diffraction peaks at 2θ = 26.78°, 41.68°, 43.94°, 50.18°, and 55.14°, corresponding to the (002), (100), (101), (102), and (004) crystal planes, respectively [32,33]. In comparison, the (002) peak of BNNS at 2θ = 26.78° is notably lower and broader, indicating that surfactant (CTAB and SDS) modification alters the crystalline structure of h-BN. Specifically, the (002) plane of BNNS exhibits a slight shift relative to pristine h-BN, which is due to the interaction between BNNS and the surfactants. Additionally, new peaks near 2θ = 25° can be attributed to the diffraction from CTAB and SDS [34].

### 4.3. Electrochemical Performance of Pristine WEP Coatings Versus BNNS/WEP Composite Coatings

**Figure 3:**
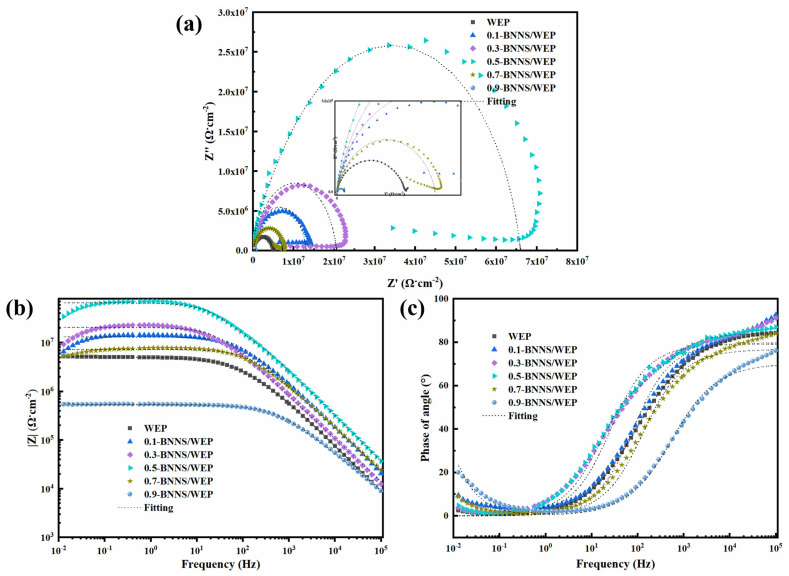
(**a**) Nyquist plots, (**b**) Bode modulus plots, and (**c**) Bode phase diagrams of pristine WEP coatings and x-BNNS/WEP composite coatings with different BNNS additions.

Figure 3 presents the EIS results for pristine WEP coatings and x-BNNS/WEP composite coatings with varying BNNS contents. In this study, we systematically evaluated the corrosion resistance of Q235 steel coated with different x-BNNS/WEP composites, each having a thickness of 50 μm (±10 μm), using EIS tests. As illustrated in Figure 3a, the corrosion resistance of the coating is positively correlated with the radius of the impedance arc; specifically, a larger arc radius indicates superior shielding performance. The impedance arc radii of the five samples are ranked as follows: 0.9-BNNS/WEP < 0.7-BNNS/WEP < 0.1-BNNS/WEP < 0.3-BNNS/WEP < 0.5-BNNS/WEP. Notably, the 0.5-BNNS/WEP sample exhibits the largest impedance arc, signifying its best shielding performance, while the 0.9-BNNS/WEP sample has the smallest arc, indicating inferior shielding properties [35]. This phenomenon may be attributed to the dispersibility and interfacial bonding strength of BNNS within the epoxy resin matrix. Optimal BNNS content can enhance the coating’s shielding effect, whereas excessive BNNS may lead to agglomeration or defects, thereby reducing coating performance. From Figure 3b, it is evident that under low-frequency conditions (|Z|_f=0.01Hz_), the impedance modulus values of the samples are as follows: 0.1-BNNS/WEP is 5.60 × 10^6^ Ω·cm^2^, 0.3-BNNS/WEP is 7.82 × 10^6^ Ω·cm^2^, 0.5-BNNS/WEP is 2.90 × 10^7^ Ω·cm^2^, 0.7-BNNS/WEP is 5.02 × 10^6^ Ω·cm^2^, and 0.9-BNNS/WEP is 5.44 × 10^5^ Ω·cm^2^. These results corroborate the findings from the Nyquist plot, indicating that 0.5 wt.% BNNS/WEP exhibits the highest impedance modulus at low frequencies, thereby demonstrating superior corrosion resistance. The impedance modulus at low frequencies primarily reflects the long-term protective capability of the coating against corrosive media, making 0.5 wt.% BNNS/WEP particularly effective in this regard. Furthermore, as illustrated in Figure 3c, 0.5 wt.% BNNS/WEP displays the largest phase angle in the high-frequency region, signifying the best barrier performance. Conversely, 0.9 wt.% BNNS/WEP has the smallest phase angle, indicating inferior blocking performance. The variation in phase angle reflects the extent to which the coating obstructs charge transfer; a larger phase angle implies more effective prevention of charge transfer, thus enhancing corrosion resistance. The largest phase angle was observed for 0.5 wt.% BNNS/WEP in the high-frequency region, which suggests its ability to provide excellent short-term protection, which is crucial for practical applications requiring immediate protection. To further investigate the performance of the epoxy-resin-based composite coating, subsequent experiments will focus on optimizing its comprehensive performance by incorporating 0.5 wt.% BNNS filler along with 0.1 wt.% FSiO_2_ and 4.9 mL FTA into the epoxy resin coating, respectively.

### 4.4. Cross-Section Morphology of Pristine WEP, 0.5-BNNS/WEP, BFS/WEP and BFT/WEP Composite Coatings

**Figure 4:**
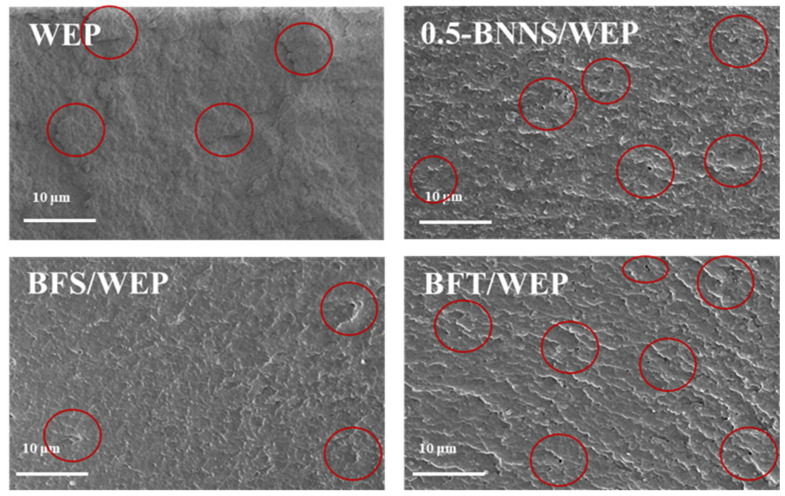
SEM images of pristine WEP coating, 0.5-BNNS/WEP, BFS/WEP and BFT/WEP composite coatings in section. The red circles in the figure indicate the presence of cracks, pores, and embedded lamellar structures in the coating.

Figure 4 presents SEM images of pristine WEP coatings and composite coatings modified with various nano-fillers. The SEM images reveal brittle fracture characteristics in the pristine WEP coating, characterized by an uneven fracture surface with numerous depressions, protrusions, significant micro-cracks, and pores. This structure indicates that the pristine WEP coating is susceptible to fracture under external forces and contains many internal defects, which adversely affect its overall performance. The cross-section of the 0.5-BNNS/WEP composite coating, prepared by incorporating 0.5 wt.% BNNS nano-filler exhibits a relatively smooth surface, with cracks at the fracture almost entirely eliminated, although some pores remain. This suggests that the BNNS nano-filler can effectively reduce defects in the pristine WEP coating, improving its density and mechanical strength, but cannot completely eliminate porosity. Further investigation revealed that the BFS/WEP composite coating, containing both 0.5 wt.% BNNS and 0.1 wt.% FSiO_2_ nano-fillers, demonstrated superior performance. The SEM image shows that cracks at the fracture have completely disappeared, pores are significantly reduced, and the coating surface exhibits an embedded lamellar structure. This multi-layer anti-corrosion barrier effectively prevents the penetration of corrosive media such as O_2_, H_2_O, and Cl^−^, primarily due to the network structure of FSiO_2,_ enhancing the coating’s density and stability. In contrast, the BFT/WEP composite coating, prepared by combining 0.5 wt.% BNNS with 4.9 mL FTA, exhibited similar properties. Although the porosity in BFT/WEP remains slightly more pronounced compared to BFS/WEP, the embedded lamellar structure of the BFT/WEP coating is more prominent, with a greater number of internal fracture layers. This is attributed to the organic network structure of FTA, which enhances the coating’s layered integrity.

### 4.5. Hydrophobic and Mechanical Properties of Pristine WEP, 0.5-BNNS/WEP, BFS/WEP and BFT/WEP Composite Coatings

**Figure 5:**
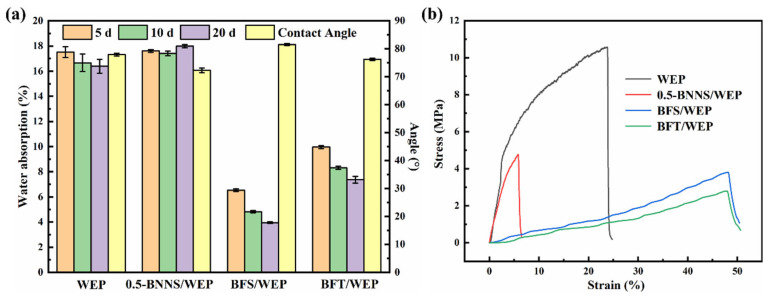
(**a**) Contact angle, water absorption tests for pristine WEP, 0.5-BNNS/WEP, BFS/WEP and BFT/WEP composite coatings. (**b**) Mechanical properties testing of pristine WEP coating, 0.5-BNNS/WEP, BFS/WEP and BFT/WEP composite coatings.

The water absorption levels of pristine WEP coating, 0.5-BNNS/WEP, BFS/WEP, and BFT/WEP composite coatings were evaluated on the 5th, 10th, and 20th day, respectively, with the results presented in Figure 5a. The water absorption rate was calculated using Formula (1). Experimental data indicate that as soaking time increases, the water absorption of the pristine WEP coating initially rises before stabilizing. This phenomenon is likely attributed to micro-cracks and other defects formed during the curing process of WEP, which allow initial water molecules to penetrate the coating, leading to increased water absorption until saturation occurs. This trend aligns with expected behavior within experimental error [36]. Research has demonstrated that an appropriate amount of nano-filler can effectively inhibit water molecule infiltration and ensure uniform dispersion within the coating. For the 0.5-BNNS/WEP composite coating, its water absorption is comparable to that of the pristine WEP coating, possibly due to the disruptive effect of exfoliated BNNS nanosheets on the coating surface, thereby compromising its barrier properties. In contrast, the BFS/WEP and BFT/WEP composite coatings exhibit significantly lower water absorption compared to both the pristine WEP and 0.5-BNNS/WEP composites. This reduction is attributed to the interaction between BNNS nanosheets and FSiO_2_ or FTA, which not only leverages the unique surface, small size, and quantum size effects of nanomaterials but also compensates for epoxy resin curing defects through their spatial network structure, thus markedly decreasing the water absorption rate. Contact angle measurements, as shown in Figure 5a, reveal contact angles of 79.3°, 72.3°, 81.9°, and 76.2° for pristine WEP, 0.5-BNNS/WEP, BFS/WEP, and BFT/WEP coatings, respectively. The synergistic effect of BNNS nanosheets with FSiO_2_ or FTA notably enhances the hydrophobicity of the coatings, thereby improving their corrosion resistance.

Subsequently, the stress–strain curves of pristine WEP coating, 0.5-BNNS/WEP, BFS/WEP, and BFT/WEP composite coatings were acquired through a servo-controlled high- and low-temperature testing apparatus. The results are shown in Figure 5b: the pristine WEP coating exhibits the highest stress value, while the BFT/WEP composite coating has the lowest stress value. Notably, the BFS/WEP and BFT/WEP composite coatings demonstrate the largest strain, whereas the 0.5-BNNS/WEP coating shows the smallest strain. The addition of BNNS nanosheets as fillers to the pure epoxy resin coating leads to stress concentration, thereby reducing both the stress and strain values of the 0.5-BNNS/WEP coating. In contrast, when FSiO_2_ and FTA are combined with BNNS as fillers in the WEP coating, the issue of reduced strain in the 0.5-BNNS/WEP composite coating is effectively mitigated. This improvement can be attributed to the spatial network structure formed by FSiO_2_ and FTA. This structure not only enhances the mechanical properties of the coating but also leads to a substantial increase in the strain of both BFS/WEP and BFT/WEP composite coatings [37].

### 4.6. Electrochemical Performance of Pristine WEP Coatings, 0.1-FSiO_2_/WEP, 4.9-FTA/WEP, 0.5-BNNS/WEP, BFS/WEP, and BFT/WEP Composite Coatings

**Figure 6:**
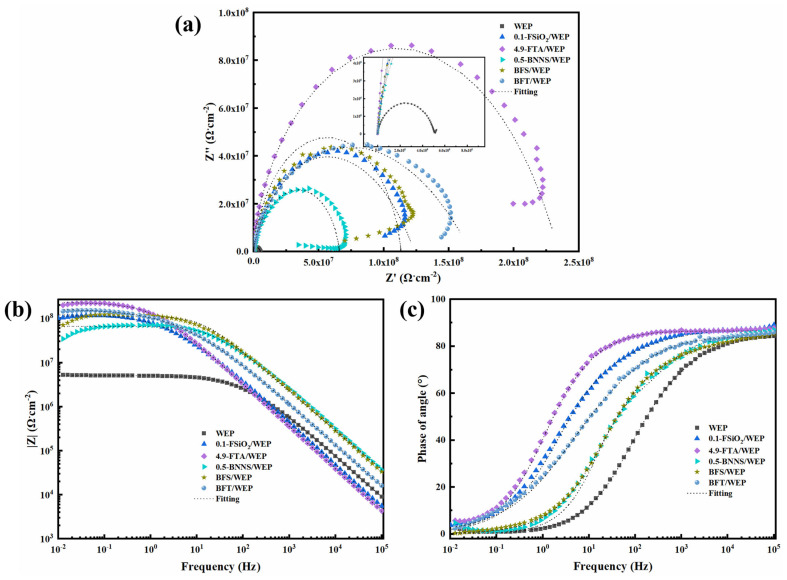
(**a**) Nyquist plots, (**b**) Bode modulus plots, and (**c**) Bode phase diagrams for pristine WEP coatings, 0.1-FSiO_2_/WEP, 4.9-FTA/WEP, 0.5-BNNS/WEP, BFS/WEP, and BFT/WEP composite coatings.

EIS was employed to investigate the corrosion resistance of Q235 steel coated with various composite coatings, each having a thickness of 50 μm (±10 μm). Figure 6 presents the EIS results for pristine WEP coatings and five different composite coatings: 0.1-FSiO_2_/WEP, 4.9-FTA/WEP, 0.5-BNNS/WEP, BFS/WEP, and BFT/WEP. The impact of corrosive media on metal substrates was assessed through EIS analysis. According to the Nyquist plot (Figure 6a), the order of impedance arc sizes is as follows: WEP < 0.5-BNNS/WEP < 0.1-FSiO_2_/WEP < BFS/WEP < BFT/WEP < 4.9-FTA/WEP. This indicates that the 4.9-FTA/WEP composite coating exhibits the largest impedance arc, while the pure WEP coating shows the smallest. The low-frequency impedance modulus (|Z|_f=0.01Hz_) can be directly obtained from the Bode modulus plot (Figure 6b). The specific values are as follows: pristine WEP (5.60 × 10^6^ Ω·cm^2^), 0.1-FSiO_2_/WEP (1.13 × 10^8^ Ω·cm^2^), 4.9-FTA/WEP (2.11 × 10^8^ Ω·cm^2^), 0.5-BNNS/WEP (2.90 × 10^7^ Ω·cm^2^), BFS/WEP (1.05 × 10^8^ Ω·cm^2^), and BFT/WEP (1.17 × 10^8^ Ω·cm^2^). Based on the Bode phase plot (Figure 6c), in the high-frequency region, the phase angle of BFT/WEP is the largest, indicating superior barrier performance. Conversely, the smallest phase angle observed for WEP suggests its inferior barrier performance.

The above phenomenon can be attributed to several factors. The addition of FSiO_2_ and FTA improves the hydrophobic performance and significantly enhances the resistance to corrosive media erosion. Secondly, FSiO_2_ and FTA have a certain bonding effect to enhance the adhesion between the coating and the metal matrix, ensuring a firm bond with the steel surface. In addition, the high dielectric constant and inherent hydrophobicity of the composite coating help to improve the anti-corrosion barrier of the composite coating [38].

### 4.7. Electrochemical Performance of 0.5-BNNS/WEP, BFS/WEP, and BFT/WEP Composite Coatings After Salt Spraying

**Figure 7:**
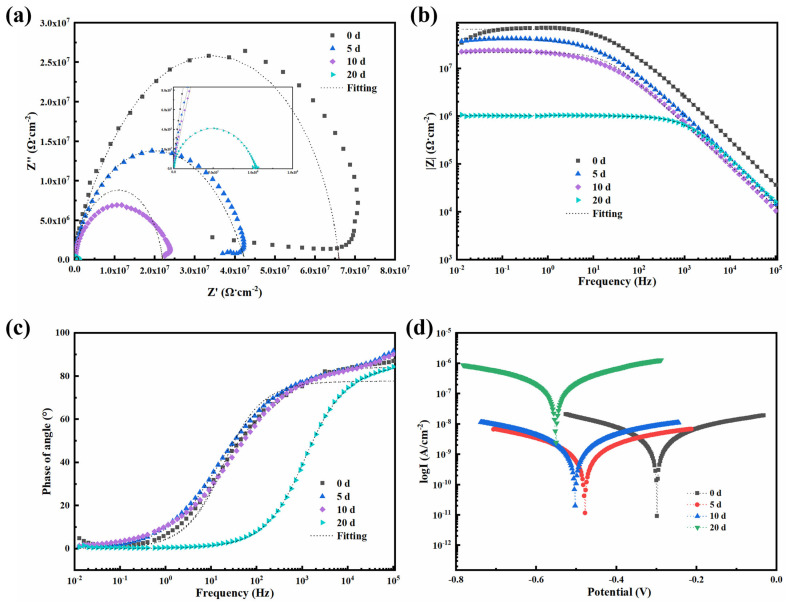
(**a**) Nyquist plots, (**b**) Bode modulus plots, (**c**) Bode phase diagrams, and (**d**) kinetic potential polarization curves for 0.5-BNNS/WEP composite coatings subjected to different corrosion times following salt spray exposure.

The effects of 0.5-BNNS/WEP, BFS/WEP, and BFT/WEP composite coatings (thickness approximately 50 μm) on the corrosion resistance of Q235 medium carbon steel were investigated using EIS in a 3.5 wt.% NaCl salt spray environment. The properties of these three composite coatings after 0, 5, 10, and 20 days of salt spray exposure were analyzed through electrochemical tests. Figure 7 illustrates (a) the Nyquist plot, (b) the Bode modulus diagram, and (c) the Bode phase diagram for the 0.5-BNNS/WEP composite coating at different salt spray durations. The fitting model is presented in Figure S2, and the fitting parameters are listed in Table S1. With the increase in salt spray exposure time, the radius of the impedance arc gradually decreases, indicating that the corrosive medium progressively penetrates the coating surface. Specifically, after 20 days of salt spray exposure, the impedance semicircle in the Nyquist plot significantly diminishes, suggesting the onset of corrosion (Figure 7a). The low-frequency impedance modulus (|Z|_f=0.01Hz_) exhibits the following trend (Figure 7b): it decreases from 2.90 × 10^7^ Ω∙cm^2^ at 0 days to 2.54 × 10^7^ Ω∙cm^2^ at 5 days, 2.18 × 10^7^ Ω∙cm^2^ at 10 days, and 1.51 × 10^6^ Ω∙cm^2^ at 20 days. This indicates that the |Z|_f=0.01Hz_ declines with increasing salt spray exposure time, thereby weakening the coating’s corrosion resistance. Additionally, the Bode phase diagram (Figure 7c) reveals that in the high-frequency region, the phase angle is largest on day 0, which indicates that the barrier property of the coating reaches its optimal state at this time. As salt spray exposure time increases, the phase angle in the high-frequency region gradually decreases, corresponding to a reduction in the coating’s barrier property. Furthermore, Figure 7d illustrates the polarization curves of the 0.5-BNNS/WEP composite coatings following various salt spray exposure durations, with the corresponding Tafel parameters summarized in Table S2. The data reveal that at day 0, the corrosion potential (E_corr_) of the 0.5-BNNS/WEP coating is −0.30 V and the corrosion current density (I_corr_) is 9.14 × 10^−12^ A/cm^2^. After 5 days, E_corr_ decreases to −0.48 V and I_corr_ increases to 1.17 × 10^−11^ A/cm^2^. By day 10, E_corr_ further drops to −0.50 V and I_corr_ rises to 2.02 × 10^−11^ A/cm^2^. At 20 days, E_corr_ reaches −0.55 V and I_corr_ escalates to 2.48 × 10^−9^ A/cm^2^. The increasingly negative corrosion potential and rising corrosion current density indicate a significant reduction in the coating’s corrosion resistance. Nevertheless, even after prolonged exposure to a salt spray environment, the corrosion resistance of the 0.5-BNNS/WEP composite coating remains largely similar to that of the untreated WEP coating, demonstrating its relatively superior long-term corrosion resistance.

**Figure 8:**
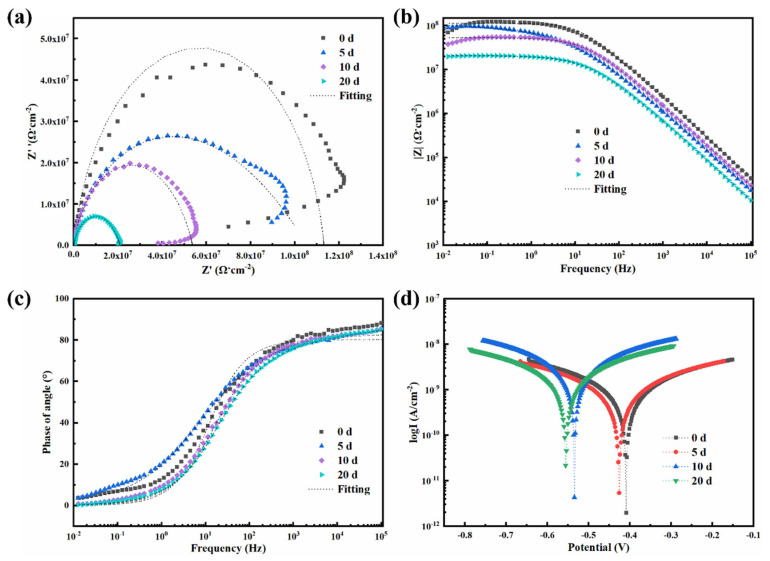
(**a**) Nyquist plots, (**b**) Bode modulus plots, (**c**) Bode phase diagrams, and (**d**) kinetic potential polarization curves for BFS/WEP composite coatings subjected to different corrosion times following salt spray exposure.

Similarly, Figure 8 displays the (a) Nyquist plot, (b) Bode modulus plot, and (c) Bode phase plot of the BFS/WEP composite coating under various salt spray exposure conditions, with the fitting parameters provided in Table S3. As the duration of salt spray exposure increases, the radius of the impedance arc exhibits a decreasing trend. Specifically, after 20 days of salt spray exposure testing, the impedance semicircle in the Nyquist plot significantly shrank, indicating that corrosion had initiated and the corrosive medium had permeated to the substrate surface. Initially (0 days), the low-frequency impedance modulus (|Z|_f=0.01Hz_) of the BFS/WEP composite coating was 1.05 × 10^8^ Ω·cm^2^. After 5 days, this value decreased to 8.72 × 10^7^ Ω·cm^2^. By 10 days, it further reduced to 3.73 × 10^7^ Ω·cm^2^; after 20 days of salt spray testing, the impedance modulus dropped to 2.01 × 10^7^ Ω·cm^2^. This trend indicates a gradual decrease in the low-frequency impedance modulus with increasing salt spray exposure time. From the Bode phase plot, it is evident that the phase angle in the high-frequency region is largest at day 0, suggesting optimal barrier performance of the coating at this stage. As salt spray exposure time increases, the phase angle in the high-frequency domain gradually decreases but remains higher than that of the pristine WEP coating, indicating that while the barrier property of the BFS/WEP coating has diminished, it still outperforms the WEP coating. Figure 8d presents the polarization curve of the BFS/WEP composite coating after various salt spray exposure durations, with Tafel parameters summarized in Table S4. The experimental data indicate that the corrosion potential (E_corr_) and corrosion current density (I_corr_) of the BFS/WEP coating exhibit changes at different time points as follows: on day 0, E_corr_ is −0.41 V and I_corr_ is 1.96 × 10^−12^ A/cm^2^; by day 5, E_corr_ decreases to −0.43 V while I_corr_ increases to 5.40 × 10^−12^ A/cm^2^. On day 10, E_corr_ further declines to −0.54 V and I_corr_ reaches 4.30 × 10^−12^ A/cm^2^. By day 20, E_corr_ drops to −0.56 V and I_corr_ significantly rises to 2.18 × 10^−11^ A/cm^2^. The increasingly negative corrosion potential and elevated corrosion current density suggest a gradual reduction in corrosion resistance. After 20 days of salt spray testing, the BFS/WEP composite coating demonstrates superior and sustained corrosion resistance in comparison with WEP coating alone.

**Figure 9:**
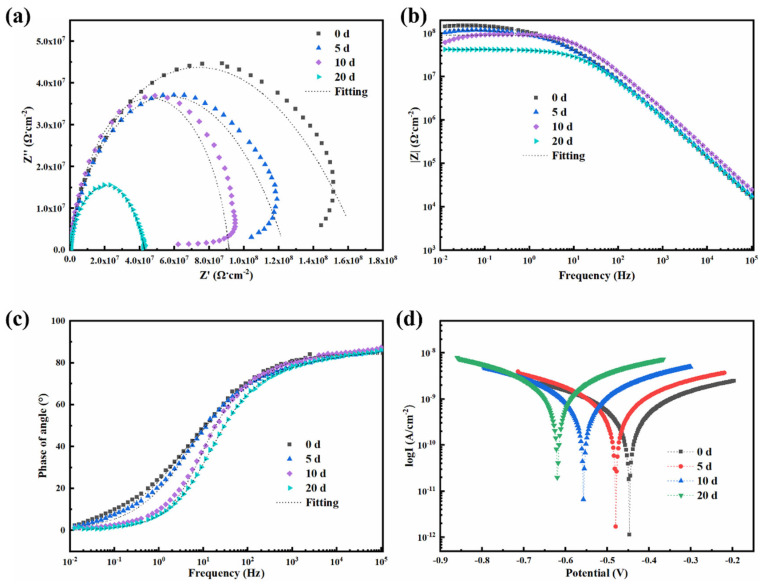
(**a**) Nyquist plots, (**b**) Bode modulus plots, (**c**) Bode phase diagrams, and (**d**) kinetic potential polarization curves for BFT/WEP composite coatings subjected to different corrosion times following salt spray exposure.

Lastly, Figure 9 presents EIS results of the BFT/WEP composite coating subjected to various salt spray exposure durations, with fitting parameters detailed in Table S5. The Nyquist plot (Figure 9a) reveals a significant reduction in the impedance semicircle after 20 days of salt spray testing, indicating the onset of corrosion and penetration of corrosive media through the coating to the substrate surface. Specifically, the low-frequency impedance modulus (|Z|_f=0.01Hz_) (Figure 9b) decreases from 1.17 × 10^8^ Ω∙cm^2^ at day 0 to 9.95 × 10^7^ Ω∙cm^2^ at day 5, further to 5.64 × 10^7^ Ω∙cm^2^ at day 10, and sharply to 4.36 × 10^6^ Ω∙cm^2^ at day 20. This progressive decline in low-frequency impedance modulus with increasing salt spray duration suggests a gradual loss of the coating’s protective efficacy, allowing more corrosive substances to penetrate and reach the metal substrate. Additionally, the Bode phase diagram (Figure 9c) shows that the phase angle in the high-frequency region is largest at day 0, indicating optimal barrier performance of the coating at this point. As salt spray exposure extends, the phase angle in the high-frequency region gradually diminishes but remains higher than that of pristine WEP coatings, suggesting that despite reduced barrier properties, the BFT/WEP composite coating still outperforms pristine WEP coatings. These findings demonstrate that the BFT/WEP composite coating retains some protective capability and can delay the corrosion process even under corrosive conditions. To further assess the corrosion resistance of the BFT/WEP composite coating, Figure 9d illustrates the polarization curves of the coating after various salt spray exposure durations, with Tafel parameters summarized in Table S6. The results show that on day 0, the E_corr_ of the BFT/WEP composite coating is −0.45 V and the I_corr_ is 1.14 × 10^−12^ A/cm^2^. After 5 days of exposure, E_corr_ decreases to −0.48 V and I_corr_ increases to 1.72 × 10^−12^ A/cm^2^. By day 10, E_corr_ further drops to −0.56 V and I_corr_ rises to 6.62 × 10^−12^ A/cm^2^. As the duration of salt spray exposure increases, both the corrosion potential becomes progressively more negative and the corrosion current density increases, indicating a gradual reduction in the coating’s corrosion resistance. After 20 days of salt spray testing, the E_corr_ value of the BFT/WEP composite coating was −0.62 V and the I_corr_ value was 1.98 × 10^−11^ A/cm^2^. These results indicate that while this composite coating exhibits superior long-term corrosion resistance in comparison with the pure WEP coating, it remains marginally inferior to the BFS/WEP coating.

**Figure 10:**
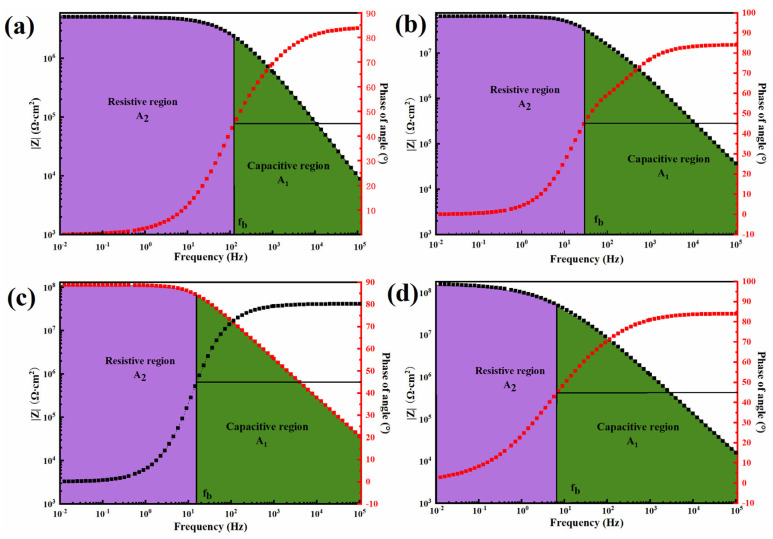
Representative Bode plots of (**a**) pristine WEP coating, (**b**) 0.5-BNNS/WEP, (**c**) BFS/WEP, (**d**) BFT/WEP composite coating, along with determining the breakpoint frequency and the corresponding capacitive (A_1_) and resistive (A_2_) regions.

Subsequently, EIS was utilized to assess the stability of the composite coating in a 3.5% NaCl electrolyte solution. The results were presented in Bode plots, which depict the frequency-dependent changes in impedance and phase angle. The region below the Bode plot can be partitioned into capacitive and resistive regions using the breakpoint frequency corresponding to a phase angle of −45°. Figure 10 illustrates the Bode plots for the pristine WEP, 0.5-BNNS/WEP, BFS/WEP, and BFT/WEP composite coatings after immersion. It is evident from the Bode plot that all coatings exhibit two distinct regions: the resistive region in the low-frequency domain and the capacitive region in the high-frequency domain. Compared to pristine WEP coatings, the resistive region of 0.5-BNNS/WEP, BFS/WEP, and BFT/WEP composite coatings progressively decreases, while the capacitive region correspondingly increases. This trend indicates that the incorporation of BNNS and its composite fillers effectively inhibits electrolyte diffusion within the coating, thereby enhancing its barrier properties. Furthermore, EIS analysis can also provide a coating damage index, which serves as an effective metric for assessing the integrity of coating systems. This index allows for the classification and evaluation of developed coating systems, confirming that BNNS-based composite fillers significantly improve the integrity of these systems. The coating damage index (D2) can be derived from the following equation [39]:

(2) $$D_{2}=\frac{A_{2}}{A_{1}}\times 100\%$$

In comparison with the pristine WEP coating, the damage index decreased after incorporating various fillers. Specifically, the damage indices of the four coating samples, in ascending order, are: BFT/WEP < BFS/WEP < 0.5-BNNS/WEP < WEP. In addition, the Bode diagram of 0.5-BNNS/WEP, BFS/WEP, and BFT/WEP composite coatings (Figure 10b–d) has a straight line with a slope of −1, which indicates the total capacitance behavior of the coating [40]. This is because the addition of compatible fillers effectively mended the micropores and cracks existing in the pure WEP coating, inhibited the diffusion of electrolytes in the coating, and reduced the probability of electrolyte contact with the substrate. This indicates that the addition of composite fillers enhances the integrity of the epoxy resin, thereby improving its anti-corrosion properties.

## 5. Conclusions

In this study, h-BN was exfoliated using surfactants CTAB and SDS, leading to the successful preparation of BNNS. The effects of varying BNNS concentrations on the properties of WEP coatings were investigated via EIS. Experimental results demonstrated that a 0.5 wt.% BNNS/WEP composite coating exhibited superior anti-corrosion performance, achieving an electrochemical impedance of 6.55 × 10^7^ Ω·cm^2^. Based on these findings, BNNS (0.5 wt.%) was further combined with FSiO_2_ (0.1 wt.%) and FTA (4.9 mL), respectively, and incorporated into WEP as fillers to explore synergistic effects. Comprehensive evaluations were conducted through contact angle measurements, water absorption tests, tensile strength assessments, and application performance testing, complemented by EIS analysis. These evaluations assessed the influence of different filler combinations (BNNS 0.5 wt.%, BNNS 0.5 wt.%/SiO_2_ 0.1 wt.%, and BNNS 0.5 wt.%/FTA 4.9 mL) on the overall performance of the epoxy resin coatings. Results indicated that compared to pure WEP coatings, the contact angles of 0.5-BNNS/WEP, BFS/WEP, and BFT/WEP coatings remained relatively unchanged, while water absorption rates were significantly reduced, demonstrating excellent anti-corrosion properties. Electrochemical impedances reached 2.90 × 10^7^, 1.05 × 10^8^, and 1.17 × 10^8^ Ω·cm^2^, respectively. After 20 days of salt spray exposure, these composite coatings maintained robust long-term corrosion resistance, with electrochemical impedances of 1.51 × 10^6^, 2.01 × 10^7^, and 4.36 × 10^6^ Ω·cm^2^, respectively. Notably, the long-term anti-corrosion performance of the BFS/WEP and BFT/WEP composite coatings exceeded that of coatings formulated with FSiO_2_ or FTA individually. In conclusion, the BFS/WEP and BFT/WEP composite coatings developed in this study exhibit superior long-term anti-corrosion properties, making them highly suitable for applications in metal anti-corrosion.

## Data Availability

The original contributions presented in this study are included in the article/Supplementary Materials. Further inquiries can be directed to the corresponding authors.

## Supplementary Materials

The following supporting information can be downloaded at: https://www.mdpi.com/article/10.3390/polym17081020/s1, Figure S1: FT-IR spectra of (a) FSiO2 and (b) FTA; Figure S2: Equivalent circuit modeling of composite coatings; Table S1: Electrochemical parameters for 0.5-BNNS/WEP composite coatings under different corrosion time obtained via simulating the impedance data based on the equivalent electrica circuits; Table S2: Polarization parameters for 0.5-BNNS/WEP composite coatings under different corrosion time; Table S3: Electrochemical parameters for BFS/WEP composite coatings under different corrosion time obtained via simulating the impedance data based on the equivalent electrical circuits; Table S4: Polarization parameters for BFS/WEP composite coatings under different corrosion time; Table S5: Electrochemical parameters for BFT/WEP composite coatings under different corrosion time obtained via simulating the impedance data based on the equivalent electrica circuits; Table S6: Polarization parameters for WEP and BFT/WEP coatings under different corrosion time.
