# Supplementary material for: Synergistic Incorporation of Boron Nitride Nanosheets and Fluoropolymers to Amplify Anti-Corrosion Attributes of Waterborne Epoxy Resin
Source: Polymers (Basel). 2025 Apr 10;17(8):1020. doi: 10.3390/polym17081020 (PMC12030106; doi:10.3390/polym17081020)
Supplement: Supplementary file 1 [file polymers-17-01020-s001.zip › polymers-3553692-supplementary.pdf]

Synergistic incorporation of boron nitride nanosheets and fluoropolymers to amplify anti-corrosion attributes of waterborne epoxy resin

Hui Ma,[1,2] Xuan Liu,[1] Xiaofeng Han,[1] Rui Yang,[1] Zhaotie Liu[1,*] and Jian Lv[2,*]

[1]Department of Chemistry and Chemical Engineering, Shaanxi University of Science and Technology, Xi'an, Shaanxi 710021, China

[2]State Key Laboratory of Fluorine & Nitrogen Chemicals, Xi'an Modern Chemistry Research Institute, Xi'an, Shaanxi 710065, China

[*]Corresponding authors.

E-mail address: ztliu@snnu.edu.cn, lujian204@263.net

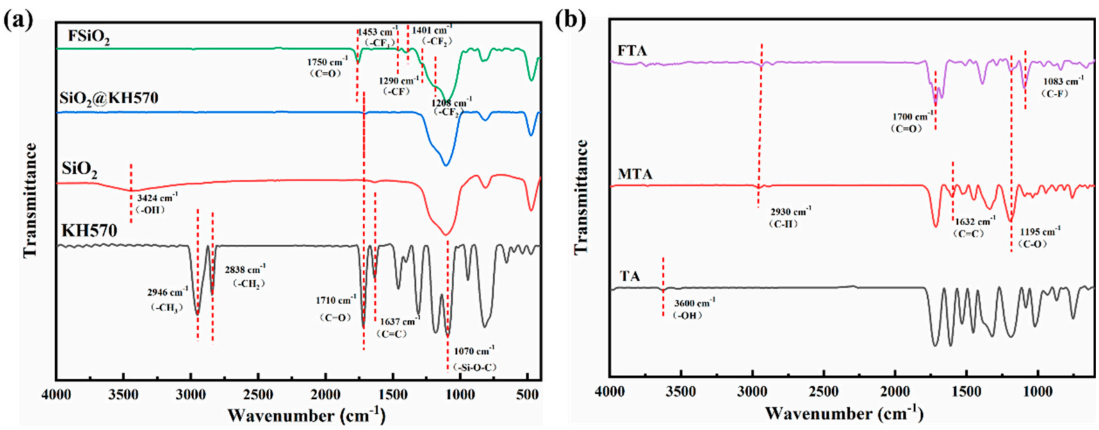

Figure S1 FT-IR spectra of (a) $FSiO_2$ and (b) FTA

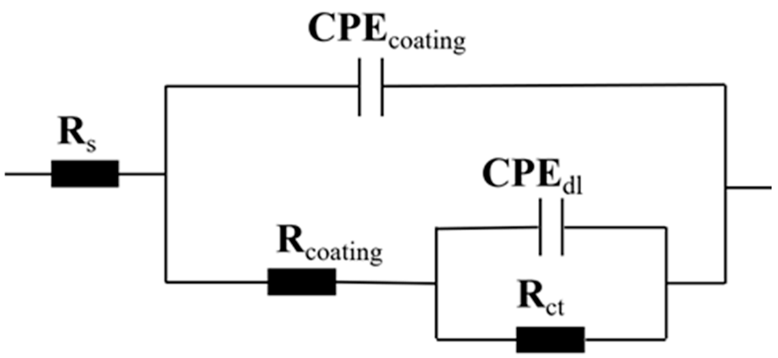

R(Q(R(QR)))

Figure S2 Equivalent circuit modeling of composite coatings

Table S1 Electrochemical parameters for 0.5-BNNS/WEP composite coatings under different corrosion time obtained via simulating the impedance data based on the equivalent electrica circuits

Sample	R_{coat} (Ω·cm^2)	CPE_{coat} (Ω^{-1}cm^{2}S^n)	n	R_{ct} (Ω·cm^2)	CPE_{dl} (Ω^{-1}cm^{2}S^n)	n
0.5-BNNS/WEP-0 d	6.554×10^{7}	1.024×10^{-10}	0.936	4.694×10^{7}	2.608×10^{-10}	0.820
0.5-BNNS/WEP-5 d	4.043×10^{7}	1.094×10^{-10}	0.953	4.214×10^{7}	1.307×10^{-9}	0.638
0.5-BNNS/WEP-10 d	2.173×10^{7}	7.592×10^{-10}	0.864	2.193×10^{7}	7.589×10^{-9}	0.973
0.5-BNNS/WEP-20 d	1.063×10^{6}	9.991×10^{-11}	0.988	1.033×10^{6}	1.071×10^{-9}	0.692

Table S2 Polarization parameters for 0.5-BNNS/WEP composite coatings under different corrosion time

Sample	E_{corr} (V)	I_{corr} (A/cm^2)	β_a (V/dec)	B_c (V/dec)
0.5-BNNS/WEP-0 d	-0.298	9.142×10^{-12}	0.199	0.159
0.5-BNNS/WEP-5 d	-0.478	1.172×10^{-11}	0.205	0.163
0.5-BNNS/WEP-10 d	-0.503	2.021×10^{-11}	0.169	0.197
0.5-BNNS/WEP-20 d	-0.551	2.475×10^{-9}	0.188	0.179

Table S3 Electrochemical parameters for BFS/WEP composite coatings under different corrosion time obtained via simulating the impedance data based on the equivalent electrical circuits

Sample	R_{coat} (Ω·cm^2)	CPE_{coat} (Ω^{-1}cm^{2}S^n)	n	R_{ct} (Ω·cm^2)	CPE_{dl} (Ω^{-1}cm^{2}S^n)	n
BFS/WEP-0 d	1.195×10^{8}	1.801×10^{-10}	0.893	1.133×10^{8}	2.227×10^{-10}	0.428
BFS/WEP-5 d	8.670×10^{7}	2.570×10^{-10}	0.922	1.047×10^{8}	2.305×10^{-9}	0.461
BFS/WEP-10 d	4.819×10^{7}	6.529×10^{-11}	0.958	5.328×10^{7}	5.563×10^{-10}	0.710
BFS/WEP-20 d	1.960×10^{7}	2.118×10^{-10}	0.974	2.097×10^{7}	1.929×10^{-9}	0.634

Table S4 Polarization parameters for BFS/WEP composite coatings under different corrosion time

Sample	E_{corr} (V)	I_{corr} (A/cm^2)	β_a (V/dec)	B_c (V/dec)
BFS/WEP-0 d	-0.409	1.961×10^{-12}	0.188	0.172
BFS/WEP-5 d	-0.425	5.400×10^{-12}	0.187	0.174
BFS/WEP-10 d	-0.543	4.296×10^{-12}	0.186	0.173
BFS/WEP-20 d	-0.556	2.184×10^{-11}	0.191	0.174

Table S5 Electrochemical parameters for BFT/WEP composite coatings under different corrosion time obtained via simulating the impedance data based on the equivalent electrica circuits

Sample	Rcoat ($\Omega\cdot cm^2$)	CPE_{coat} ($\Omega^{-1}cm^2S^n$)	n	Rct ($\Omega\cdot cm^2$)	CPE_{dl} ($\Omega^{-1}cm^2S^n$)	n
BFT/WEP-0 d	1.417×10^{8}	2.416×10^{-10}	0.935	1.606×10^{9}	1.521×10^{-9}	0.505
BFT/WEP-5 d	9.950×10^{7}	1.599×10^{-10}	0.957	3.256×10^{7}	1.196×10^{-9}	0.574
BFT/WEP-10 d	8.868×10^{7}	1.504×10^{-10}	0.938	3.074×10^{7}	1.638×10^{-10}	0.754
BFT/WEP-20 d	4.318×10^{7}	7.515×10^{-10}	0.702	4.296×10^{7}	1.011×10^{-10}	0.987

Table S6 Polarization parameters for WEP and BFT/WEP coatings under different corrosion time

Sample	E_{corr} (V)	I_{corr} (A/cm^2)	β_a (V/dec)	B_c (V/dec)
BFT/WEP-0 d	-0.447	1.137×10^{-12}	0.186	0.179
BFT/WEP-5 d	-0.480	1.715×10^{-12}	0.192	0.168
BFT/WEP-10 d	-0.557	6.623×10^{-12}	0.187	0.177
BFT/WEP-20 d	-0.620	1.975×10^{-11}	0.192	0.182
